# Safety and short-term efficacy of preoperative FOLFOX therapy in patients with resectable esophageal squamous cell carcinoma who are ineligible for cisplatin

**DOI:** 10.1007/s10388-022-00951-4

**Published:** 2022-09-01

**Authors:** Toru Kadono, Shun Yamamoto, Toshiharu Hirose, Go Ikeda, Akihiro Ohara, Mai Itoyama, Kazuki Yokoyama, Yoshitaka Honma, Taiki Hashimoto, Shigeki Sekine, Koshiro Ishiyama, Junya Oguma, Hiroyuki Daiko, Ken Kato

**Affiliations:** 1 Department of Head and Neck, Esophageal Medical Oncology, National Cancer Center Hospital, 5-1-1 Tsukiji, Chuo-ku, Tokyo, 104-0045 Japan; 2 Course of Advanced Clinical Research of Cancer, Juntendo University Graduate School of Medicine, Tokyo, Japan; 3 Department of Diagnostic Pathology, National Cancer Center Hospital, Tokyo, Japan; 4 Department of Esophageal Surgery, National Cancer Center Hospital, Tokyo, Japan

**Keywords:** Esophageal neoplasms, Neoadjuvant therapy, Oxaliplatin, 5-Fluorouracil, Leucovorin

## Abstract

**Background:**

The standard preoperative treatment for resectable locally advanced esophageal squamous cell carcinoma (LAESCC) in Japan is docetaxel, cisplatin (CDDP), and 5-fluorouracil. However, patients with renal or cardiac dysfunction and elderly patients are ineligible for a CDDP-containing regimen because of toxicities. Oxaliplatin, leucovorin, and 5-fluorouracil (FOLFOX) therapy has less renal toxicity than CDDP-containing regimens and does not require hydration. However, there are limited data on preoperative FOLFOX therapy in these patients.

**Methods:**

This retrospective study analyzed patients with resectable LAESCC who were aged ≥ 75 years or had renal or cardiac dysfunction and received preoperative FOLFOX between 2019 and 2021. FOLFOX was administered every 2 weeks for 3 or 4 cycles and was followed by surgery. Adverse events associated with chemotherapy, the complete resection (R0) rate, relative dose intensity (RDI), and histopathological response were evaluated.

**Results:**

Thirty-five patients were eligible. Median age was 77 (range 65–89) years; 68.6% were aged ≥ 75 years, 74.3% had renal dysfunction, and 17.1% had cardiac dysfunction. The RDI was 70.2% and 87.1% for bolus and continuous intravenous 5-fluorouracil, respectively and 85.2% for oxaliplatin. The most common grade ≥ 3 adverse events were neutropenia (60.0%) and leucopenia (28.6%). Two patients (5.7%) had febrile neutropenia and grade 3 pneumonia. Thirty-one patients underwent surgery. The R0 resection rate was 87.1%, and there was no histopathological evidence of residual tumor in 16.1%. There were no treatment-related deaths.

**Conclusions:**

Preoperative FOLFOX had a manageable safety profile and showed favorable short-term efficacy in patients with resectable LAESCC who were ineligible for CDDP-containing treatment.

## Introduction

Esophageal cancer (EC) has the sixth highest mortality rate of all cancers worldwide [1]. EC is divided histologically into two main types: squamous cell carcinoma and adenocarcinoma. In 2013, 87.8% of patients with EC in Japan had esophageal squamous cell carcinoma (ESCC) and 61.2% of all patients with EC underwent esophagectomy [2].

In Western countries, the standard treatment for resectable locally advanced EC is preoperative chemoradiotherapy followed by surgery plus postoperative nivolumab based on the results of the CROSS and CheckMate 577 trials [3, 4]. However, in Japan, the standard treatment is preoperative docetaxel, cisplatin (CDDP), and 5-fluorouracil (DCF) followed by surgery based on the results of the JCOG1109 trial, which showed preoperative DCF to be superior to CDDP plus 5-fluorouracil (CF) in terms of survival [5]. Nevertheless, preoperative CF remains a treatment option because preoperative DCF is more toxic than CF, especially in elderly or vulnerable patients [6]. Moreover, patients with renal or cardiac dysfunction are ineligible for these CDDP-containing treatments due to the renal toxicity of CDDP and the need for high-volume hydration. In clinical practice, we have used other treatments for patients with resectable locally advanced esophageal squamous cell carcinoma (LAESCC) who are not eligible for CDDP, including preoperative chemotherapy (e.g., nedaplatin plus 5-fluorouracil) followed by surgery, surgery alone, definitive chemoradiotherapy, or radiotherapy alone [7–16]. However, no standard treatment has been established for these patients because they have been excluded from the clinical trials.

Oxaliplatin, leucovorin, and 5-fluorouracil (FOLFOX) therapy is an optional regimen for EC. FOLFOX is less emetogenic and has less renal toxicity than CDDP-containing regimens and does not require hydration [17–19]. In the PRODIGE5/ACCORD17 trial, patients who received definitive chemoradiotherapy that included FOLFOX had a trend of less nausea and renal toxicity (all grades, 49% and 1%, respectively) than those who received CF (all grades, 61% and 5%, respectively) [17] without a decrease in efficacy. Moreover, FOLFOX was shown to have efficacy comparable with that of CF as palliative chemotherapy in the E-DIS trial [20]. However, there are no data on the safety and efficacy of preoperative FOLFOX in patients who are ineligible for CDDP.

This study assessed the safety and efficacy of preoperative FOLFOX in patients with resectable LAESCC who were not eligible for CDDP.

## Methods

### Patients

This retrospective study analyzed patients with advanced EC who received preoperative FOLFOX at the National Cancer Center Hospital between October 2019 and October 2021. The main selection criteria were as follows: histologically proven esophageal squamous cell carcinoma; cT1N1-3M0, cT2-3N0-3M0, or cT1-3N0-3M1 (M1 disease limited to supraclavicular lymph node metastasis) (Union for International Cancer Control, TNM Classification of Malignant Tumors, 8th edition); age ≥ 75 years, renal dysfunction (creatinine clearance < 60 mL/min), or cardiac dysfunction (ejection fraction ≤ 50% on cardiac ultrasound, past history of heart failure, or poorly controlled arrhythmia); and no prior therapy for EC. Preoperative FOLFOX (oxaliplatin 85 mg/m^2^, leucovorin 200 mg/m^2^, and a bolus of 5-fluorouracil 400 mg/m^2^ on day 1 followed by 5-fluorouracil 2400 mg/m^2^ over 46 h) was administered intravenously every 2 weeks for 3 or 4 cycles according to the patients’ overall condition as determined by the attending oncologist. The study was approved by the Institutional Review Board of the National Cancer Center, Japan (approval number: 2020–287) and conducted in accordance with the ethical principles outlined in the Declaration of Helsinki. Because of the retrospective nature of this study, informed consent was not obtained from each patient. Patient consent was obtained using an opt-out method.

### Assessments

Computed tomography (CT) scans were obtained before initiation of preoperative FOLFOX, before cycle 2, and before surgery. The CT schedule after surgery was determined by the surgeons. Laboratory investigations were performed at the initiation of each treatment cycle and before surgery.

The primary outcomes were adverse events during preoperative chemotherapy and histopathological response. Adverse events were graded according to the Common Terminology Criteria of Adverse Events (CTCAE) version 5.0. Histopathological response was classified according to the proportion of tumor tissue that degenerated or became necrotic using the grading system outlined in the Japanese Classification of Esophageal Cancer, 11th edition [21]: grade 0, no part of tumor affected; grade 1a, less than one-third affected; grade 1b, between one-third and two-thirds affected; grade 2, between two-thirds and entire tumor affected; and grade 3, no residual tumor. Pathological T0 or Tis N1-3 were treated as T1 N1-3.

The secondary outcomes were the relative dose intensity (RDI) of preoperative chemotherapy, response to preoperative chemotherapy, complete resection (R0) rate, progression-free survival (PFS), and overall survival (OS). The RDI was calculated on the scheduled dose intensity divided by the actual delivered dose intensity. The scheduled period in patients received 3 cycles was 6 weeks and in patients received 4 cycles was 8 weeks. The response to chemotherapy was evaluated by CT based on the Response Evaluation Criteria in Solid Tumors (RECIST), version 1.1 [22]. PFS was defined as the time from the start of preoperative FOLFOX until progression or presence of new lesions or death, or was censored in cases with survival without progression. OS was defined as the time from the start of preoperative FOLFOX until death or was censored in surviving patients at the last follow-up visit. Survival was estimated using Kaplan–Meier curves.

## Results

### Patient characteristics

Forty-four patients received preoperative FOLFOX. Nine patients were excluded because they had histology other than squamous cell carcinoma (*n* = 6), distant lymph node metastasis (*n* = 2), or prior therapy for EC (*n* = 1), leaving 35 patients eligible for inclusion in the study. The characteristics of the study participants at the start of chemotherapy are shown in Table 1. Median age was 77 (range 65–89) years. The patients were considered ineligible for CDDP for the following reasons: age ≥ 75 years (68.6%), renal dysfunction (74.3%), and cardiac dysfunction (17.1%). Median creatinine clearance in patients with renal dysfunction was 45.0 (range 26.4–56.1) mL/min.

**Table 1:** Characteristics of 35 patients with resectable locally advanced esophageal squamous cell carcinoma who received preoperative FOLFOX

	*n* (%)
Age (years)	
Median (range)	77 (65–89)
Sex	
Male/female	25 (71.4)/10 (28.6)
ECOG PS	
0/1	14 (40.0)/21 (60.0)
Clinical TNM stage	
T1b/T2/T3	4 (11.4)/5 (14.3)/26 (74.3)
N0/N1/N2 /N3	8 (22.9)/18 (51.4)/9 (25.7)/0 (0)
M0/M1	34 (97.1)/1 (2.9)
Clinical stage	
I/II/III/IVB	4 (11.4)/10 (28.6)/20 (57.1)/1 (2.9)
Reasons for selecting FOLFOX	
Renal dysfunction	26 (74.3)
Age ≥ 75 years	24 (68.6)
Cardiac dysfunction	6 (17.1)
Comorbidity	
Hypertension	26 (74.3)
Cerebral infarction	8 (22.9)
Diabetes mellitus	9 (25.7)
Smoking	24 (68.6)
Alcohol consumption	27 (77.1)

*ECOG PS* Eastern Cooperative Oncology Group performance status, *FOLFOX* oxaliplatin, leucovorin, and 5-fluorouracil, *TNM* tumor-node-metastasis

**Fig. 1:**
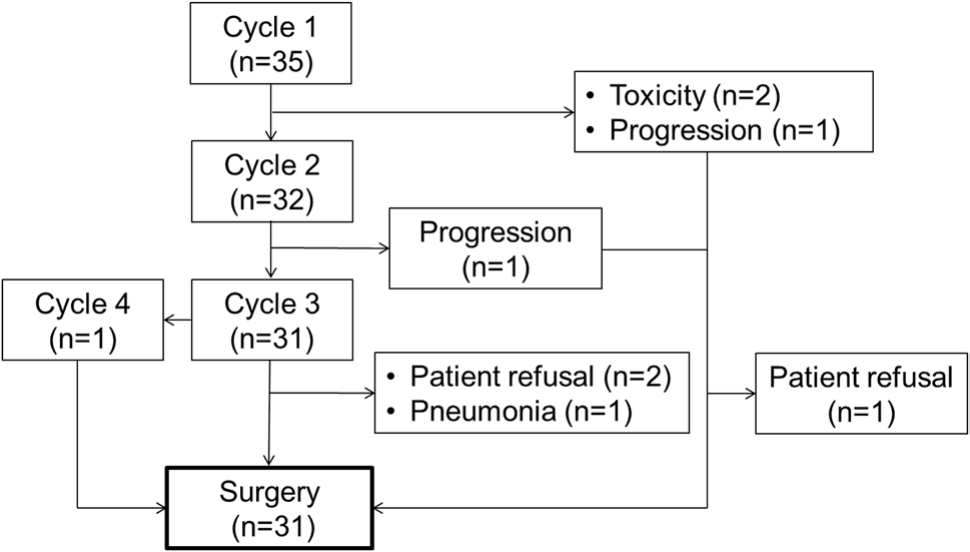
Treatment flow for all patients who were received preoperative FOLFOX therapy

The treatment details are shown in Fig. 1. Thirty-five patients received 1 cycle of preoperative chemotherapy and three discontinued after 1 cycle because of grade 3 febrile neutropenia (*n* = 1), grade 3 neutropenia (*n* = 1), or disease progression (*n* = 1). One patient discontinued preoperative chemotherapy after 2 cycles because of disease progression. Thirty-one patients (88.6%) received 3 cycles and 1 received 4 cycles. Three of the patients who received three or 4 cycles of preoperative chemotherapy did not undergo esophagectomy (patient refusal, *n* = 2; grade 3 pneumonia as a toxicity, *n* = 1). Three of 4 patients who discontinued preoperative chemotherapy underwent esophagectomy and 1 declined surgery. Finally, 31 patients (88.6%) underwent esophagectomy.

Four patients (11.4%) had a dose reduction during the initial cycle because of decision by attending oncologist (*n* = 2), renal dysfunction (*n* = 1), or a low platelet count (*n* = 1). The dose was reduced between the second and third cycles in 13 patients (37.1%) because of adverse events (neutropenia, *n* = 7; elevated creatinine, *n* = 2; febrile neutropenia, *n* = 1; thrombocytopenia, *n* = 1; diarrhea, *n* = 1) or because it was considered necessary in the opinion of the attending oncologist (*n* = 1). The RDI was 70.2% and 87.1% for bolus and continuous intravenous 5-fluorouracil, respectively and 85.2% for oxaliplatin.

### Safety of preoperative FOLFOX

Thirty-five patients who received at least 1 cycle of preoperative chemotherapy were assessed for adverse events (Fig. 1). The most common grade ≥ 3 adverse events were neutropenia (60.0%) and leucopenia (28.6%). Two patients (5.7%) developed febrile neutropenia. Sixteen (76.2%) of the 21 patients who developed neutropenia had renal dysfunction. Granulocyte colony-stimulating factor (G-CSF) was administered as secondary prophylaxis in 9 patients (25.7%). Two patients (5.7%) had grade 3 pneumonia and 1 patient (2.9%) had grade 3 stomach pain. Grade ≥ 3 nausea and decreased appetite occurred in 1 patient (2.9%). Elevated creatinine was seen in 2 patients (5.7%; from 0.89 to 1.18 mg/dL in one and from 0.86 to 1.01 mg/dL in the other). No treatment-related deaths occurred during preoperative chemotherapy (Table 2).

**Table 2:** Adverse events during preoperative chemotherapy

Grade (CTCAE v5.0)	1 (%)	2 (%)	3 (%)	4 (%)	Any grade (%)	Grade ≥ 3 (%)
Hematological						
Leukopenia	5 (14.3)	9 (25.7)	9 (25.7)	1 (2.9)	24 (68.6)	10 (28.6)
Neutropenia	4 (11.4)	3 (8.6)	13 (37.1)	8 (22.9)	28 (80.0)	21 (60.0)
Anemia	18 (51.4)	10 (28.6)	2 (5.7)	0 (0)	30 (85.7)	2 (5.7)
Thrombocytopenia	20 (57.1)	8 (22.9)	1 (2.9)	0 (0)	29 (82.9)	1 (2.9)
Non-hematological						
Nausea	6 (17.1)	3 (8.6)	1 (2.9)	0 (0)	10 (28.6)	1 (2.9)
Decreased appetite	6 (17.1)	2 (5.7)	1 (2.9)	0 (0)	9 (25.7)	1 (2.9)
Fatigue	6 (17.1)	0 (0)	0 (0)	0 (0)	6 (17.1)	0 (0)
Constipation	2 (5.7)	4 (11.4)	0 (0)	0 (0)	6 (17.1)	0 (0)
Mucositis	3 (8.6)	1 (2.9)	0 (0)	0 (0)	4 (11.4)	0 (0)
Peripheral neuropathy	2 (5.7)	0 (0)	0 (0)	0 (0)	2 (5.7)	0 (0)
Febrile neutropenia	−	−	2 (5.7)	0 (0)	2 (5.7)	2 (5.7)

*CTCAE v5.0* Common Terminology Criteria of Adverse Events version 5.0

### Surgical outcomes and efficacy

Thirty-one of the 35 patients underwent esophagectomy. Two-stage surgery was performed in 7 patients. Open esophagectomy was performed in 1 patient (3.2%), laparoscopic esophagectomy in 22 (71.0%), mediastinoscopic esophagectomy in 5 (16.1%), and robot-assisted esophagectomy in 3 (9.7%). Postoperative complications were pneumonia (*n* = 11, 35.5%), recurrent laryngeal nerve paralysis (*n* = 8, 25.8%), anastomotic leakage (*n* = 5, 16.1%) pleural effusion (*n* = 3, 9.7%), and acute heart failure (*n* = 1, 3.2%). There were no surgery-related deaths.

**Fig. 2:**
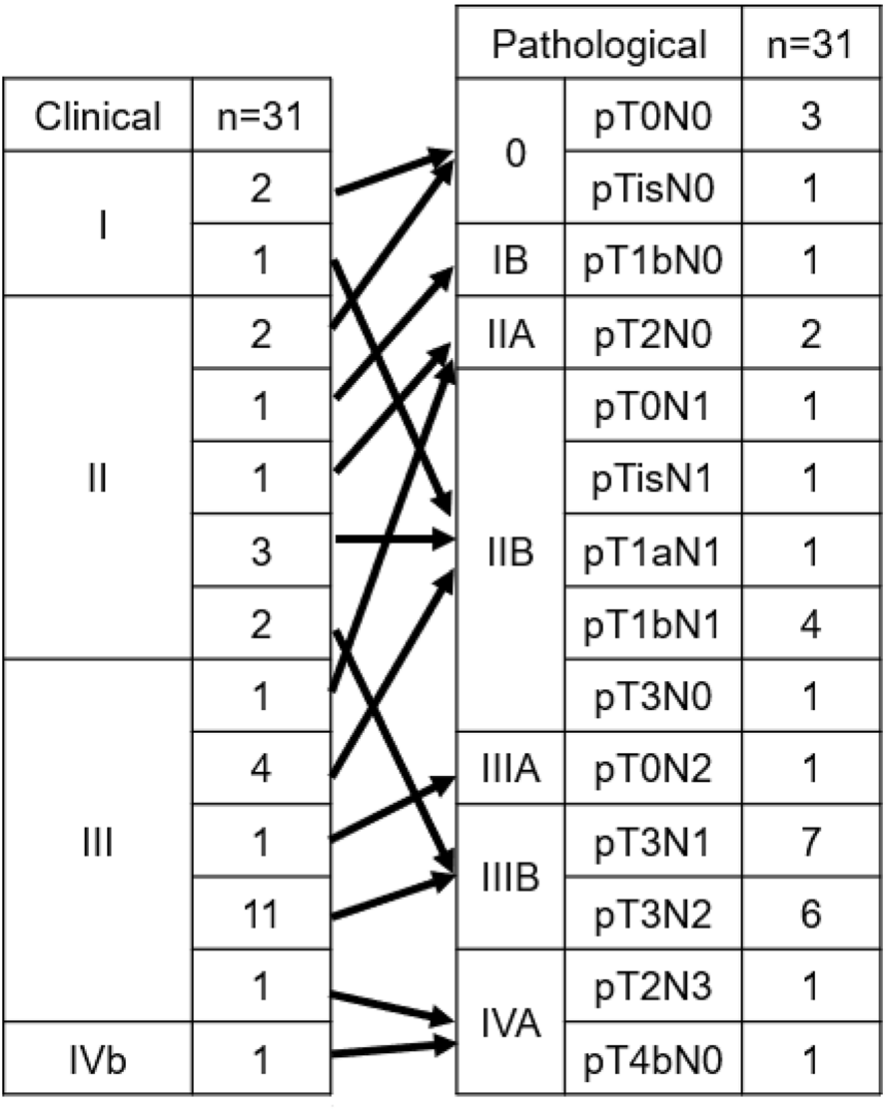
Relationship between clinical stage and pathological stage in 31 patients. Pathological T0-is N+ was treated as pT1 N+. Downstaging was possible in 32.3% patients

**Fig. 3:**
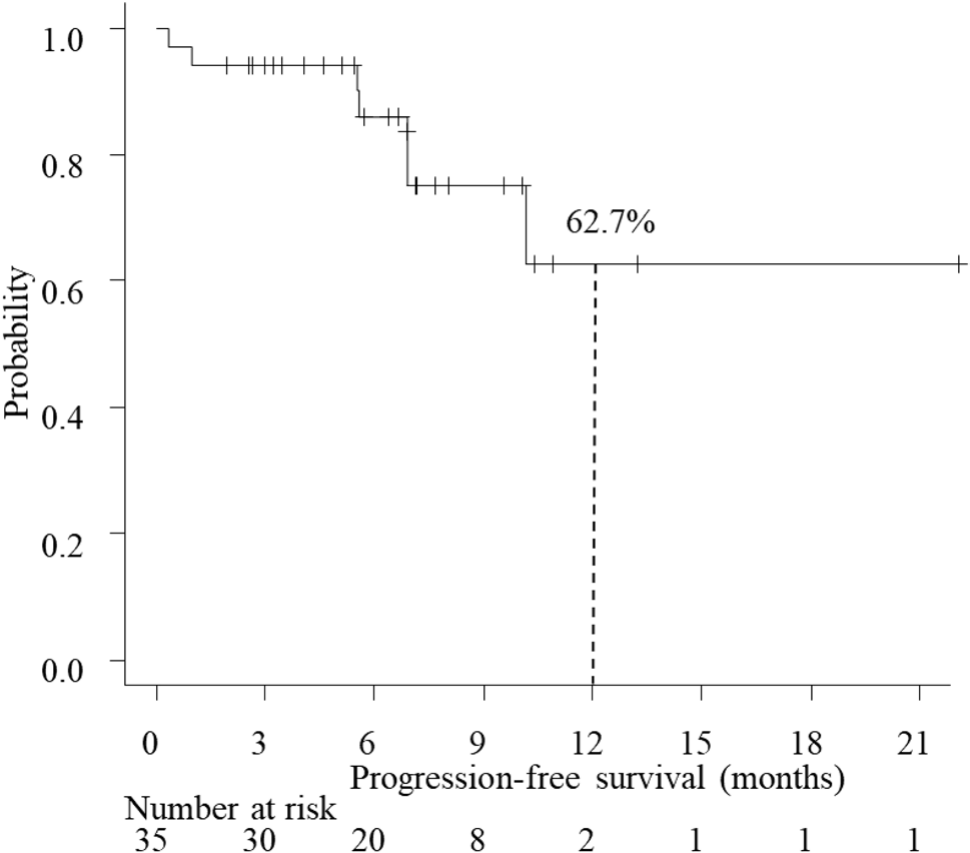
Kaplan–Meier curves for progression-free survival (PFS). The 1-year PFS rate was 62.7%

**Fig. 4:**
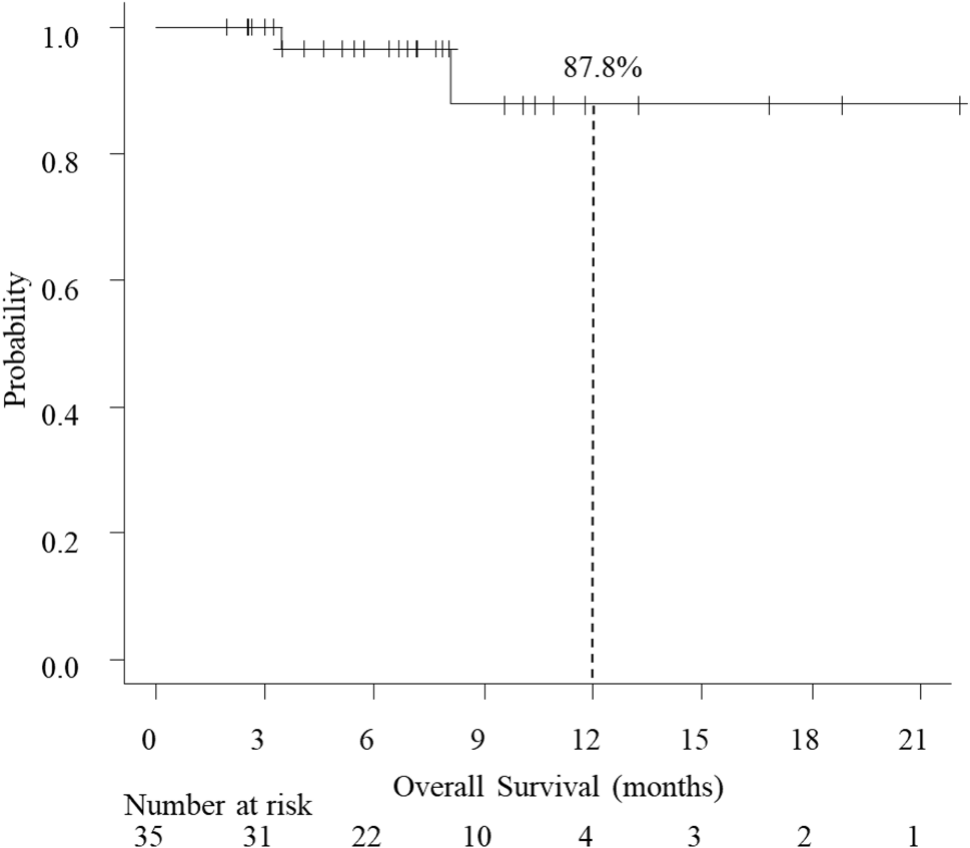
Kaplan–Meier curves showing overall survival (OS). The 1-year OS rate was 87.8%

The R0 resection rate was 87.1% (27/31). The histopathological response rate was 0% for grade 0, 64.5% (20/31) for grade 1a, 0% for grade 1b, 19.4% (6/31) for grade 2, and 16.1% (5/31) for grade 3. The baseline distributions of clinical and pathological stages are shown in Fig. 2. Only 6 of the 35 patients had measurable target lesions; 1 (16.7%) had a complete clinical response to preoperative FOLFOX, 2 (33.3%) showed a partial response, 1 (16.7%) had stable disease, and 2 (33.3%) had progressive disease. The objective response rate was 50.0%. Neither median PFS nor OS was reached after a median follow-up duration of 6.9 (range 1.9–22.1) months. The 1-year PFS and OS rates were 62.7% (95% CI 30.9–83.1%) and 87.8% (95% CI 54.9–97.2%), respectively (Figs. 3 and 4).

## Discussion

In this study, patients with resectable LAESCC who were not eligible for CDDP showed a favorable histopathological response to preoperative FOLFOX therapy. Although there was a high frequency of severe neutropenia, non-hematological adverse events were mild. In particular, any renal or gastrointestinal toxicity was tolerable.

Based on the results of the JCOG1109 trial, the standard treatment for LAESCC is DCF chemotherapy followed by surgery [5]. In other studies, 66.7% of patients who received preoperative DCF therapy developed nausea and 35.7% developed elevated creatinine of any grade [5, 23]. These vulnerable patients who cannot tolerate DCF are considered for preoperative CF. However, nausea was reported to occur in 72.4% and elevated creatinine of any grade in 23.7% of patients who received preoperative CF [24]. Therefore, even preoperative CF may be too toxic for elderly patients and patients with renal or cardiac dysfunction. A single-center retrospective study in Japan found that survival was shorter in elderly patients who underwent esophagectomy after CF than in those who underwent esophagectomy without preoperative chemotherapy [7]. The same study, vulnerable elderly patients who received preoperative CF were more likely to develop severe adverse events, including renal dysfunction, esophageal fistula, and aspiration pneumonia. Furthermore, only half of the patients who received preoperative CF were able to complete their planned course. However, the findings of another retrospective study indicated that elderly patients had worse survival than their younger counterparts because they were less likely to receive preoperative chemotherapy [8]. These data suggest that preoperative chemotherapy helps to prolong survival but that preoperative CF is inappropriate for patients who are ineligible for CDDP.

In the present study, adverse events that occurred during preoperative FOLFOX therapy were manageable. Nausea of any grade was observed in 28.6% of patients and grade ≥ 3 nausea was observed in 2.9%. This mild emetogenic toxicity was also reflected in the low frequencies of decreased appetite of any grade (25.7%) and grade ≥ 3 (2.9%). Decreased appetite and nausea can lead to malnutrition, and weight loss before esophageal surgery is associated with a higher risk of mortality [25, 26]. Therefore, the mild toxicity of FOLFOX in terms of nausea and decreased appetite might contribute to longer survival compared with surgery after CF and help patients maintain a good overall condition. Furthermore, preoperative FOLFOX caused renal toxicity in only 2 patients. However, grade ≥ 3 neutropenia occurred in 60.0% of patients and febrile neutropenia occurred in 5.7%. The likely explanation for this high rate of neutropenia is that our study included elderly patients and patients with renal dysfunction, both of which are known to be risk factors for neutropenic complications during chemotherapy [27]. Indeed, in our study, most cases of grade ≥ 3 neutropenia occurred in patients with renal dysfunction. G-CSF was used as secondary prophylaxis in 25.7% of the patients in this study but not for primary prophylaxis in any of the patients. However, G-CSF should be considered for primary prophylaxis in patients with renal dysfunction. Postoperative complications have been observed more frequently after preoperative FOLFOX therapy than after preoperative CF, especially pneumonia and recurrent laryngeal nerve paralysis (35.5% and 25.8%, respectively, after preoperative FOLFOX; 10.3% and 15.1% after preoperative CF) [5]. We suspected this was also the case in our study because it included older patients. However, there were no in-hospital deaths.

In this study, the grade 3 histopathological response rate was 16.1% for preoperative FOLFOX, which is higher than the rate of 2.2% reported for preoperative CF [5], although this is a cross-trial comparison. Moreover, downstaging was possible in 32.3% of our patients (Fig. 2) and the R0 resection rate was 87.1%, which is comparable with the rates of 90.3% for preoperative CF and 94.5% for DCF [5].

To our knowledge, this study is the first to evaluate the safety and efficacy of preoperative FOLFOX therapy. However, it had some limitations. First, the study was performed retrospectively at a single institution. Second, although we defined age ≥ 75 years, renal dysfunction (creatinine clearance < 60 mL/min), and cardiac dysfunction (ejection fraction ≤ 50% by cardiac ultrasound, past histology of heart failure, or poorly controlled arrhythmia) as criteria for selecting patients who were ineligible for CDDP in this study, the treatment strategy was decided at the discretion of each medical oncologist without clear criteria for eligibility for CDDP. Third, the survival data are immature because of the short follow-up duration and occurrence of few events. However, we found a high histopathological complete response rate, which is associated with a good prognosis [28]. Further investigation of the longer-term efficacy is needed. Fourth, approximately a quarter of the patients in the study underwent mediastinoscopic or robot-assisted esophagectomy. Although these procedures might be less invasive, their impact on recurrence and survival is unclear. Fifth, the appropriate number of cycles of preoperative FOLFOX therapy has not been established. We think that three cycles (six weeks) might be suitable because the number of cycles of preoperative doublet CF therapy is two cycles (six weeks) based on the results of JCOG9907 trial [6]. Moreover, we have considered that three cycles of preoperative FOLFOX therapy is suitable because of the toxicity. Nine patients developed grade ≥ 3 neutropenia between the second and third cycles (2 patients discontinued after 1 cycle and 7 patients had a dose reduction); however, grade ≥ 3 neutropenia was seen in 28 patients during preoperative chemotherapy. It meant that 19 patients (54.3%) developed grade ≥ 3 neutropenia after cycle 3. The RDI is expected to decline if four cycles is administered.

In conclusion, preoperative FOLFOX was well tolerated and demonstrated favorable short-term efficacy in patients with LAESCC who were not eligible for CDDP.
